# Eliminating the invading extracellular and intracellular FnBp^+^ bacteria from respiratory epithelial cells by autophagy mediated through FnBp-Fn-Integrin α5β1 axis

**DOI:** 10.3389/fcimb.2023.1324727

**Published:** 2024-01-09

**Authors:** Meiqi Meng, Jiachao Wang, Hongru Li, Jiao Wang, Xuan Wang, Miao Li, Xue Gao, Wenjian Li, Cuiqing Ma, Lin Wei

**Affiliations:** ^1^ Department of Immunology, Key Laboratory of Immune Mechanism and Intervention on Serious Disease in Hebei, Hebei Medical University, Shijiazhuang, China; ^2^ Clinical Laboratory, the Second Hospital of Hebei Medical University, Hebei Key Laboratory of Laboratory Medicine, Shijiazhuang, China

**Keywords:** autophagy, Integrin α5β1, FnBp, S100A8, FnBp^+^ bacteria

## Abstract

**Background:**

We previously found that the respiratory epithelial cells could eliminate the invaded *group A streptococcus* (GAS) through autophagy induced by binding a fibronectin (Fn) binding protein (FnBp) expressed on the surface of GAS to plasma protein Fn and its receptor integrin α5β1 of epithelial cells. Is autophagy initiated by FnBp^+^ bacteria via FnBp-Fn-Integrin α5β1 axis a common event in respiratory epithelial cells?

**Methods:**

We chose *Staphylococcus aureus* (*S. aureus/S. a*) and *Listeria monocytogenes* (*L. monocytogenes/L. m*) as representatives of extracellular and intracellular FnBp^+^ bacteria, respectively. The FnBp of them was purified and the protein function was confirmed by western blot, viable bacteria count, confocal and pull-down. The key molecule downstream of the action axis was detected by IP, mass spectrometry and bio-informatics analysis.

**Results:**

We found that different FnBp from both *S. aureus* and *L. monocytogenes* could initiate autophagy through FnBp-Fn-integrin α5β1 axis and this could be considered a universal event, by which host tries to remove invading bacteria from epithelial cells. Importantly, we firstly reported that S100A8, as a key molecule downstream of integrin β1 chain, is highly expressed upon activation of integrin α5β1, which in turn up-regulates autophagy.

**Conclusions:**

Various FnBp from FnBp^+^ bacteria have the ability to initiate autophagy via FnBp-Fn-Integrin α5β1 axis to promote the removal of invading bacteria from epithelial cells in the presence of fewer invaders. S100A8 is a key molecule downstream of Integrin α5β1 in this autophagy pathway.

## Introduction

Multiple bacteria, including traditional intracellular bacteria (e.g., *L. monocytogenes*) and extracellular bacteria, such as GAS and *S. aureus*, can invade epithelial or endothelial cells and escape from phagocytosis by immune cells or antibiotic attack (Py et al., 2007; Mitchell et al., 2018; Xie et al., 2020; Caire et al., 2022; Rodrigues Lopes et al., 2022), which may be responsible for recurrent or chronic bacterial infections that continue to threaten human health; therefore, new therapeutic breakthroughs are urgently needed.

The cytoplasm is the battlefield for survival between host and pathogen (Patterson et al., 2021). Autophagy is a common response of host cells to pathogen exposure, usually, autophagy is beneficial to host cells to eliminate pathogen. Autophagy is a highly conserved fundamental intracellular biological process that maintains cellular homeostasis by recycling defective organelles or proteins (Shibutani and Yoshimori, 2014; Kuo et al., 2018; Nakatogawa, 2020). In recent years, accumulating evidence has shown that autophagy plays a complementary and connecting role in the clearance of bacteria by both innate and adaptive immunity (Deretic and Levine, 2009; Krakauer, 2019). There are different categories of autophagy, including macroautophagy, microautophagy, chaperone-mediated autophagy, and xenophagy (Shahnazari and Brumell, 2011; Schille et al., 2018). After bacterial infection, the host defense mechanism can perform xenophagy (Lu et al., 2017; Kwon and Song, 2018; Sorbara et al., 2018). During this process, bacteria, in particular intracellular pathogens, can be engulfed directly into the cytoplasm by a bilayer membrane vesicle called autophagosome which then fuses with the lysosome for degradation. Alternately, bacteria can be surrounded by a monolayer membrane structure called LC3-associated phagosome (LAP) (Gibson et al., 2021; Prajsnar et al., 2021). Related literature points out xenophagy is an important intracellular innate immune protective mechanism (Neumann et al., 2016; Kemper and Hensel, 2023).

Whether the outcome of the confrontation is beneficial to the host or the pathogen depends on whether the pathogen is more invasive or the host is more defensive when infection occurs (Shahnazari and Brumell, 2011). In our previous work, we confirmed that M1 GAS initiates autophagy which results in pathogen elimination in respiratory epithelial cells through its structural protein FbaA, a member of the FnBp. FnBp binds first to plasma protein Fn which then binds receptor integrin expressed on the epithelial cells (Tang et al., 2012). Many bacteria like GAS express FnBps (hereinafter termed as FnBp^+^ bacteria) and utilize Fn to bind its receptor α5β1 to invade epithelial or endothelial cells(Ma et al., 2009; Josse et al., 2017; Speziale et al., 2019), in order to evade phagocytosis. These bacteria include intracellular bacteria, such as Mycobacterium tuberculosis (TB), *Mycobacterium bovis* (BCG), *Mycobacterium leprae* (Mle), *Neisseria meningitidis* (NME), *Neisseria gonorrhoeae* (NGO), *Borrelia burgdorferi* (Bbu), *Yersinia pestis* (Ype), *L. monocytogenes*, as well as extracellular bacteria like GAS, *S. aureus* and *Lactococcus lactis* (Lla). However, while integrin α5β1 expressed on epithelial cells is an invasive gateway for pathogens, but also it is a part of a signaling axis that induces autophagy, which is confirmed by our previous study. Extending our previous work, we asked if integrin α5β1 acts as a receptor that induces autophagy through FnBp binding to Fn and to integrin α5β1. We further asked if the initiation of autophagy via the FnBp-Fn-integrin α5β1 axis could be considered a common event. To address these questions, we purified the major FnBps expressed on the surface of two common bacteria, *S. aureus* (extracellular FnBp^+^ bacteria) and *L. monocytogenes* (intracellular FnBp^+^ bacteria), and found that these proteins could, indeed, induce xenophagy. We also confirmed that the autophagy induced by these FnBp^+^ bacteria were mediated through the FnBp-Fn-integrin α5β1 axis. Moreover, to the best of our knowledge, we are the first to identify S100A8, which inactivates mTOR thus inducing upregulated autophagy, is a key downstream regulatory molecule of integrin α5β1.

## Materials and methods

### Bacterial culture

*S. aureus* (ATCC 26001) and *L. monocytogenes* (ATCC 19115) were stored at – 80°C in our laboratory. Cryopreserved bacteria were inoculated onto Luria-Bertani (LB) or brain heart infusion (BHI) agar plates at 37°C and incubated for 24 h. An *S. aureus* single colony was transferred to 3 ml of LB at 37°C and shaken at 220 rpm overnight. Single colony of *L. monocytogenes* was transferred to 3 ml of BHI medium at 28°C and shaken at 220 rpm overnight to amplify the bacteria.

### Cells

The human respiratory epithelial cell line Hep2 (obtained from The Institute of Basic Medical Sciences of the Chinese Academy of Medical Sciences, China) was cultured in RPMI-1640 medium (Gibco) supplemented with 100 U/ml penicillin and 100 U/ml streptomycin (Solarbio), 10 mM HEPES (AMRESCO0511; Biosharp), and 10% fetal bovine serum (Biological Industries) at 37°C with 5% CO_2_ in an incubator.

### Infection

Hep2 cells were seeded at 3×10^5^/well in a 6-well plate containing complete RPMI-1640 medium. Before infection, cells were washed by PBS three times, and medium was changed to RPMI-1640 without antibiotics. As for *S. aureus*, Hep2 cells were cocultured with bacteria inoculated at a multiplicity of infection (MOI) of 10:1 for 2 h at 37°C with 5% CO_2_ in an incubator. The optimal MOI for *L. monocytogenes* was 20. To kill adherent extracellular bacteria, cells were washed three times with PBS and then incubated with fresh RPMI-1640 containing 100 g/ml gentamicin at 37°C for 2h. Finally, Hep2 cells were collected at specific time points for Western blot analysis.

### shRNA knockdown

Hep2 cells stably expressing specific shRNA against ATG5 or luciferase gene were established by being transduced with lentiviral particles expressing specific shRNA. Lentiviral particles were packaged by transfecting 293T cells with pSIF-H1-copGFP shRNA Expression Lentivectors (System Biosciences) and packaging vectors using Lipofectamine 2000 according to the manufacturer’s instructions. The sequences used in the shRNA targeting ATG5 and luciferase (the control shRNA) were as follows: 5’-TCATGGAATTGAGCCAATGTT-3’ and 5’-CTTACGCTGAGTACT TCGA-3’. Western blotting was performed to determine knockdown efficiency.

### siRNA

Before transfection, Hep2 cells were seeded wells of a 6-well plate. When the Hep2 cells grew to 60% confluence, they were transiently transfected with the corresponding siRNA by Lipo2000. After 48 h, silencing efficiency was determined with Western blotting.

The following siRNAs were used:

Fn siRNA, sense, 5-GUCCUGUCGAAGUAUUUAUTT-3;
antisense, 5-AUAAAUACUUCGACAGGACTT-3;
integrin α5 siRNA, sense, 5-CACCCGAAUUCUGGAGUAUTT-3;
antisense, 5-AUACUCCAGAAUUCGGGUGTT-3;
Integrin β1 siRNA, sense, 5-GCACCAGCCCAUUUAGCUATT-3;
antisense, 5-UAGCUAAAUGGGCUGGUGCTT-3;

### Expression and purification of the S.a-FnBpA protein and L.m-FbpA protein

Expression of 6×His Tag S.a-FnBpA and 6×His Tag L.m-FbpA protein was performed on vector pET-28a-transformed *E. coli* BL21 following induction with isopropyl-D-thiogalactopyranoside (IPTG) (0.1 mM; Merck). The 6×His Tag S.a-FnBpA and 6×His Tag L.m-FbpA proteins were purified by glutathione Sepharose 4B (GE Healthcare) and the SKL method, respectively.

### Western blotting

Cells were collected and lysed with radio immunoprecipitation assay lysis buffer (P0013; Beyotime) containing phenylmethylsulfonyl fluoride (BL507A; Biosharp) and phosphatase inhibitors (P1260; Solarbio) on ice for 30 min. The components in the supernatant (denatured at 100°C in sample buffer) were separated using SDS-PAGE and transferred to 0.45mm or 0.22mm polyvinyl difluoride (PVDF) membranes (IPVH00010; Millipore). The PVDF membranes then were blocked with 5% nonfat milk for 1 h and incubated overnight with primary antibodies at 4°C. Subsequently, the PVDF membranes were washed with Tris-buffered saline-Tween 20 and incubated with the corresponding secondary antibody for 1 h at room temperature. Finally, the proteins were detected with *Western Lightning* ^™^ *Plus* ECL reagent (NEL104001EA; PerkinElmer) and detected with a Synoptics Syngene bioimaging instrument (R114075; Synoptics).

### Antibodies and reagents

For Western blot analysis, the following antibodies were used: anti-LC3B (2775; Cell Signaling Technology), anti-SQSTM1/P62 (5114; Cell Signaling Technology), anti-ATG5 (CY5766; Abways), anti-fibronectin (ab32419; Abcam), anti-integrin α5 (CY5979; Abways), anti-integrin β1 (CY5469; Abways), anti-phospho-mTOR (Ser2448) (AF3308; Affinity Biosciences), anti-mTOR (AF6308; Affinity Biosciences), and anti-glyceraldehyde-3-phosphate dehydrogenase (GAPDH) (5174; Cell Signaling Technology). Horseradish peroxidase-labeled goat anti-rabbit (ASS1009; Abgent) secondary antibodies were used. The transfection reagents were Lipofectamine 2000 (11688-019; Invitrogen) and Lipo6000™(Beyotime). The immunoprecipitation (IP) reagent was included in the Pierce classic magnetic IP/co-IP kit (88804; Thermo Scientific).

### Immunofluorescence

Hep2 cells were seeded in wells of a 12-well plate and transfected with pBABEpuroEGFP-LC3 plasmids using Lipofectamine 2000 for 24 h. Cells were then stimulated by *S. aureus* or S.a-FnBpA protein for 6h. As for *L. monocytogenes* and L.m-FbpA protein, the period of stimulation was 4h. After washing with PBS, cells were fixed in 4% paraformaldehyde for 20 min and blocked in 1% bovine serum albumin for 1 h. Cells were then incubated with the corresponding primary antibody overnight at 4°C. After four rinses, the secondary antibody was used at a suitable concentration for 1 h. *4*′,*6*-*diamidino*-*2*-*phenylindole* (DAPI) was used to stain cell nuclei. Finally, cells were visualized with an Olympus confocal fluorescence microscope.

### Immunoprecipitation

IP assays were performed according to the instructions of the Pierce classic magnetic IP/co-IP kit. Briefly, cells were lysed in specific buffer on ice for 30 min. Supernatant protein was then incubated overnight with the corresponding antibody on a rotator at 4°C. The next day, Pierce protein A/G beads were washed with specific buffer three times. Supernatant-antibody mixture and beads were then co-incubated on a rotator at room temperature for 4h, washed with lysis buffer and PBS, and boiled for 10 min. Samples were subjected to SDS-PAGE and Western blot analysis, and target proteins were detected using the corresponding antibodies.

### Transmission electron microscopy

Autophagosomes induced by *S. aureus* and *L. monocytogenes* in the Hep2 cells were analyzed under a Hitachi 7500 transmission electron microscope with a small modification. Briefly, Hep2 cells were seeded in wells of a 6-well plate and infected with *S. aureus* and *L. monocytogenes* at their optimal MOI for 2h. After washing with PBS three times, cells were incubated for an additional 6h. Cells were then trypsinized and collected by centrifuging at 1,000 rpm for 5 min and fixed overnight with 2.5% glutaraldehyde in 0.1 M sodium cacodylate buffer at 4°C. Subsequently, the fixed cells were post-fixed with 1% osmic acid, dehydrated stepwise with ethanol, and embedded in epoxy resin. Ultrathin sections were cut using a Leica ultramicrotome and stained with uranyl acetate and lead citrate. Cells were imaged using a Hitachi 7500 transmission electron microscope at an 80-kV acceleration voltage. The procedure described above was performed at the Electron Microscope Center of Hebei Medical College.

### H&E staining

Histology of pathogen-infected mouse lung tissue was analyzed using hematoxylin eosin staining (H&E). In brief, mice were anesthetized using isoflurane in a biosafety cabinet. *S. aureus* and *L. monocytogenes* were delivered intranasally (i.n.) (3×10^8^ CFU in 50μl of PBS). After 24 h, mouse lungs were fixed with 4% polyformaldehyde, embedded in paraffin, cut into 5mm-thick sections, and stained with H&E. The slides were examined by light microscopy. The degree of inflammation in the alveolar tissue, peri-bronchial and perivascular spaces were graded as follows: 0: normal;1: increased thickness of the inter alveolar septa (IAS) by edema and cell infiltration;2: increased thickness of IAS with presence of luminal cell infiltration; 3: abundant luminal cell infiltration; 4: inflammatory patches formed. In each tissue section, 10 alveolar tissue fields, 10 airways, and 10 blood vessels were observed and analyzed. Grading was performed by pathologists from Hebei Medical University.

### Cells and lung CFU determination

Hep2 cells were infected with *S. aureus* or *L. monocytogenes* (MOI=10, 20 or 100) for 6 h at 37°C in 5% CO_2_. After washing with pre-cooled PBS three times, the infected monolayers were dispersed by the addition of 0.25% trypsin and then lysed by dilution with sterile water for 12 min. The number of bacterial CFUs released from the lysed cells was determined by plating lysates on LB/BHI agar at 37°C for 24 h. Mice were infected with pathogen, and lungs were subsequently aseptically isolated and weighed. Then lungs were homogenized in RPMI-1640 (containing Gentamicin) and lysed as described above with sterile water. The number of bacterial CFUs released from the lysed lung cells was determined by plating the lysates on LB/BHI agar at 37°C for 24 h.

### Animal experiments

C57 mice (6-week-old females for *S. aureus* infection and 12-week-old females for *L. monocytogenes* infection) were purchased from the Experimental Animal Center of Hebei Medical University. The Laboratory Animal Quality Certificate number is 1811002. All experimental procedures were performed in compliance with institutional animal welfare guidelines and were carried out according to the criteria outlined in the Guide for the Care and Use of Laboratory Animals and with approval of the Animal Care and Use Committee of Hebei Medical University. Mice were maintained in an animal facility (temperature-controlled individual ventilated cages) under a 12-h light/dark cycle and were fed standard chow and sterile tap water.

### Atg5-KO mice

Atg5^flox/flox^ mice were provided by RIKEN BRC through the National Bio-Resource Project of MEXT, Japan, and kindly donated by Quan Chen, Institute of Zoology, Chinese Academy of Sciences. Specific Atg5^flox/cre^ mice were generated by mating Atg5^flox/flox^ mice with Sftpc-cre mice (purchased from ViewSolid Biotech, Beijing, China). Intraperitoneal injection of tamoxifen (Sigma Aldrich) was administered to inducible cre-driver lines. The injection dose was determined by weight using approximately 75 mg/kg of body weight to determine tamoxifen dose which was administered via intraperitoneal injection once every 24 h for a total of 5 consecutive days. For Cre characterization work, a 7-day waiting period is mandatory between the final injection and necropsy/histological analysis.

### Statistical analysis

SPSS statistical software (version 18.0) was used for statistical analysis. The data are expressed as the means ± standard deviation (SD). The significance between two groups was determined using unpaired *t* test. A *P* value of <0.05 was considered significant. All experiments were performed in triplicate or more replicates.

### Ethics approval and consent to participate

All methods were carried out in accordance with relevant guidelines and regulations.

## Results

### *S. aureus* could induce autophagy both *in vivo* and *in vitro*

**Figure 1:**
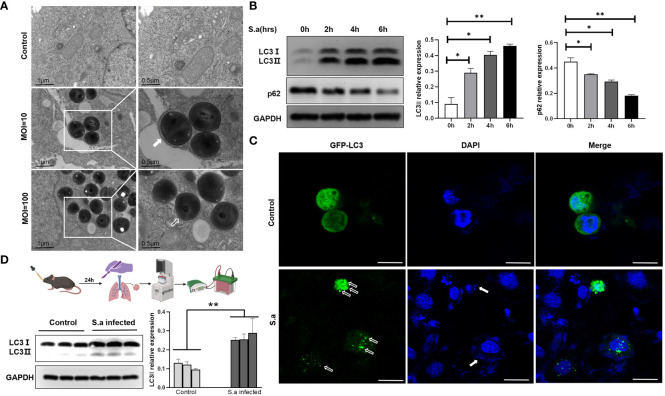
*S. aureus* can induce autophagy, both *in vivo* and *in vitro.* **(A)** Presence of autophagosomes or LAP in *S. aureus*-infected Hep2 cells was observed by transmission electron microscopy. Under varied infection conditions, the membrane structure around the intracellular invading bacteria is different. White frames mark the areas scrutinized. White arrow points to autophagy bilayer vesicles, whereas hollow arrow points to a monolayer membrane structure called LAP. **(B)** Under the condition of lower MOI (MOI=10), the expression of LC3II and p62 was analyzed by Western blotting of proteins from *S. aureus*-infected Hep2 cells. The method of the LC3II relative WB quantifications presented is LC3 II/I: GAPDH. The method of the p62 relative WB quantifications presented is p62: GAPDH. N = 3 repeats and the quantifications are means ± SD fold-change relative to control conditions for a given protein after normalization with GAPDH. **(C)** After Hep2 cells were transfected with the pBABE-puro-EGFP-LC3 plasmid for 24 h and then infected with *S. aureus*, the host cells stained with DAPI and the presence of fluorescent EGFP-LC3 were determined by confocal laser scanning microscopy. White hollow arrow points to LC3 puncta, whereas solid white arrow points to invaded bacteria. Scale bar, 25 μm. **(D)** Experimental mice were i.n. infected with PBS or *S. aureus*. After 24 hours, the mice were sacrificed and the tissue protein was extracted by lung grinding (route showed in legend). LC3II expression in the lungs of each experimental group was analyzed by Western blotting. Unpaired *t* test was used for data analysis. *P<0.05, **P<0.01.

Some research groups reported that *S. aureus* can be phagocytized and then decomposed through classical autophagy after invading cells (Zang et al., 2020). Nevertheless, some researchers have found that this pathogen also exhibits the tenacity needed to “use” autophagosomes, which otherwise serve to engulf bacteria, in order to replicate and multiply in cells (Wang et al., 2021). Here, respiratory epithelial cell Hep2 was treated by different multiplicity of infection (MOI). Under the electron microscope, we observed that a large number of *S. aureus* invaded cells and that an obvious monolayer membrane structure appeared around them in the condition of high MOI (MOI=100), a condition described above as LAP. In this case, the cells quickly die owing to bacterial infection. However, when we simulated the colonization of *S. aureus* in the human respiratory tract by infecting Hep2 cells with a lower MOI (MOI=10), a typical bilayer membrane structure was formed, enclosing the invading bacterium (**Figure 1A**). In order to confirm the observed bilayer membrane structure as an autophagosome that formed in the autophagy pathway, we harvested the cells and extracted total proteins to measure the expression of autophagy marker proteins LC3 and p62. The results showed that the protein level of LC3II increased and that of p62 decreased with the passage of time (**Figure 1B**). Additionally, Hep2 cells with EGFP-LC3-overexpression were infected with *S. aureus* for 6h, and the results showed that LC3 protein with GFP green fluorescence appeared obvious spot-like aggregation distribution in the cytoplasm compared with the control group (**Figure 1C**), suggesting that a low bacterial load of *S. aureus* can induce the production of complete autophagic flux. To further verify these results *in vivo*, we extracted tissue proteins from the lungs of mice following *S. aureus* challenge via the intranasal route (i.n.) and found that the protein level of LC3II was significantly higher than that of the control group (**Figure 1D**). The above results indicate that the autophagy process does, indeed, occur in the presence of a bilayer membrane structure which are identified as an autophagosome and that complete autophagy flow is induced by *S. aureus* with low MOI *in vivo* and *in vitro*.

### *L. monocytogenes* could induce autophagy *in vivo* and *in vitro*

**Figure 2:**
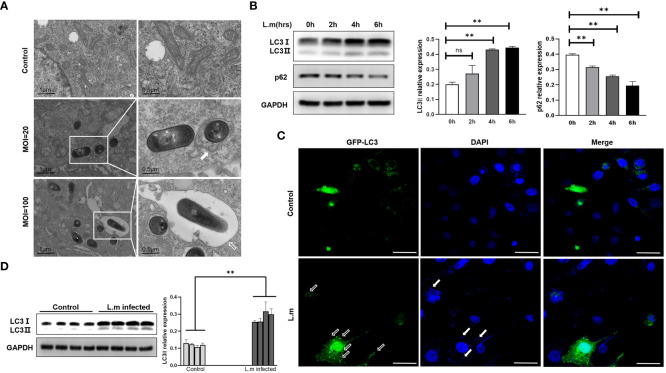
*L. monocytogenes* could induce autophagy *in vivo* and *in vitro* **(A)** The *L. monocytogenes* that invaded Hep2 cells was observed under electron microscopy. The types of phagosomes formed under varied infection conditions were different. The white frames mark areas scrutinized. White arrow points to autophagy bilayer vesicles, whereas hollow arrow points to a monolayer membrane structure called LAP. **(B)** Hep2 cells were infected with *L. monocytogenes* at a MOI of 20, and then the total proteins were collected to determine the changes of autophagy marker proteins with time gradient. N = 3 repeats and the quantifications are means ± SD fold-change relative to control conditions for a given protein after normalization with GAPDH. **(C)** Hep2 cells were transfected with pBABE puro-EGFP-LC3 plasmid with green fluorescence by Lipo2000. Twenty-four hours later, Hep2 cells were infected with *L. monocytogenes* up to the experimental time point and then observed by confocal microscopy. LC3 protein with GFP green fluorescence showed obvious spot-like aggregation and distribution in the cytoplasm of infected Hep2 cells. White hollow arrow points to LC3 puncta, whereas solid white arrow points to invaded bacteria. Scale bar, 25 μm. **(D)** Experimental mice were i.n. infected with PBS or *L. monocytogenes* nasal drops. After 24 hours, the mice were sacrificed and the tissue protein was extracted by lung grinding (Same as in legend1). LC3II expression in the lungs of each experimental group was analyzed by Western blotting. Unpaired *t* test was used for data analysis. *P<0.05, **P<0.01. ns, no significance.

After verifying the ability of *S. aureus*, an extracellular bacterium, to induce autophagy, we further asked whether traditional intracellular bacteria could induce a similar phenomenon? Accordingly, we chose *L. monocytogenes*, a classical facultative intracellular pathogen, to infect Hep2 cells with different infection plurals (MOI=20 or 100). Consistently, the tendency of *L. monocytogenes* in infecting epithelial cells was very similar to that of *S. aureus*. That is, more monolayers were formed around invading bacteria in the case of high MOI, whereas more bilayer membrane structures surrounded intracellular bacteria following infection with low MOI (**Figure 2A**). Next, we measured the expression tendency of autophagy marker proteins at different time points after *L. monocytogenes* infection. Similar to *S. aureus*, results confirmed that the protein level of LC3II increased and that of p62 decreased with the passage of time under the condition of low MOI (**Figure 2B**). LC3 protein with GFP green fluorescence also showed obvious spot-like aggregation distribution in the cytoplasm of Hep2 cells in the infected group compared with the control group (**Figure 2C**). Additionally, we infected WT C57BL/6N mice i.n. with *L. monocytogenes* and found that the protein level of LC3II in the lungs of mice in the infected group was significantly higher than that of the control group (**Figure 2D**). The above results indicate that *L. monocytogenes* can induce autophagy in epithelial cells with low MOI *in vivo* and *in vitro*.

### Autophagy is responsible for eliminating invading bacteria from cells

**Figure 3:**
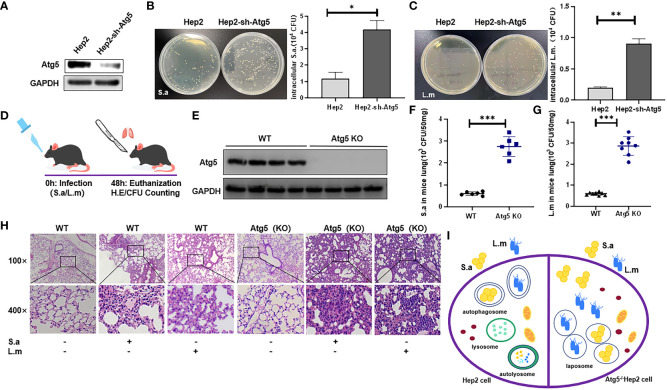
Autophagy may be related to the clearance of intracellular bacteria. **(A)** Western Blot analysis of the expression of Atg5 protein in Sh-atg5- transfected cells or Sh-ctrl cells. **(B, C)** WT Hep2 and Atg5-knockdown cells were infected with *S. aureus* or *L. monocytogenes* (MOI=20) for 6 h. The intracellular viable bacteria were counted in the cells and then analyzed. **(D)** WT and ATG5 (KO) mice were infected i.n. with *S. aureus* (3×10^8^ CFU) or *L. monocytogenes* (5×10^8^ CFU) to establish a pulmonary infection model. **(E)** ATG5 (KO) mice were established and verified by Western blot. **(F, G)** The number of viable bacteria (CFU) was analyzed in the lung of mice infected i.n. with *S. aureus* or *L. monocytogenes*. **(H)** H&E staining analysis of pulmonary inflammation in WT and ATG5 (KO) mice after *S. aureus* or *L. monocytogenes* infection. The degree of inflammation in the alveolar tissue, peri-bronchial and perivascular spaces were graded as following (from left to right): 0; 2; 2; 0; 4; 4. **(I)** Schematic diagram of the different fates of invading bacteria in WT cells and Atg5-defective cells. Unpaired *t* test was used for data analysis. *P<0.05, **P<0.01, ***P<0.005.

We found that autophagy could be induced by these two common bacteria, but we wonder whether autophagy could promote the removal of the invading pathogens? Therefore, Atg5-knockdown Hep2 cells (autophagy-knockdown cells) and WT Hep2 cells (**Figure 3A**) were infected with *S. aureus* or *L. monocytogenes* (MOI=20) for 6 hours, and viable bacteria in the infected cells were counted (**Figures 3B, C**). Results showed that the number of viable bacteria in Atg5-knockdown cells was significantly higher than that in WT Hep2 cells, suggesting that knockdown of Atg5 can block the clearance of viable bacteria in cells and that the clearance process of bacteria in Hep2 cells may be related to autophagy.

Further, ATG5 (KO) mice generated from Sftpc-cre mice and Atg5^flox/flox^ mice were subjected to intranasal bacterial infection. At the end of time point, mice were sacrificed, and lungs were harvested, following the specified institutional ethical requirements (see Methods) (**Figure 3D**). Results showed that the number of viable bacteria in the lungs of ATG5 (KO) mice (**Figure 3E**) infected with either *S. aureus* or *L. monocytogenes* was significantly higher than that of WT mice (**Figures 3F, G**), indicating that knockout of Atg5 in mice is associated with the decreased ability of eliminating viable bacteria in the lung tissues and that the clearance process of pathogens in lungs of FnBp^+^ bacteria-infected mice is related to autophagy. Compared with the PBS group, lung of WT C57BL/6N mice infected with *S. aureus* or *L. monocytogenes* showed severe lung inflammation by H&E staining, along with enhanced lymphocyte infiltration and incrassated alveolar septa. Compared with WT C57BL/6N mice, ATG5 (KO) mice showed more severe inflammatory response in the lungs with deeper infiltration of inflammatory cells, large amount of bleeding in the alveoli, severe thickening of alveolar septa and an increased area of inflammatory lesions following infection with either *S. aureus* or *L. monocytogenes* (**Figure 3H**). These results suggest that autophagy is a biological process which is involved in the host elimination of invading bacteria, both *in vivo* and *in vitro*.

Taken together, under the condition of physiological colonization, *S. aureus* or *L. monocytogenes* invading cells could be eliminated by intracellular autophagy process, which is not achieved in cells or animals with defective autophagy (**Figure 3I**).

### FnBp on the surface of pathogens can promote the eliminating ability of invading bacteria by initiating autophagy

**Figure 4:**
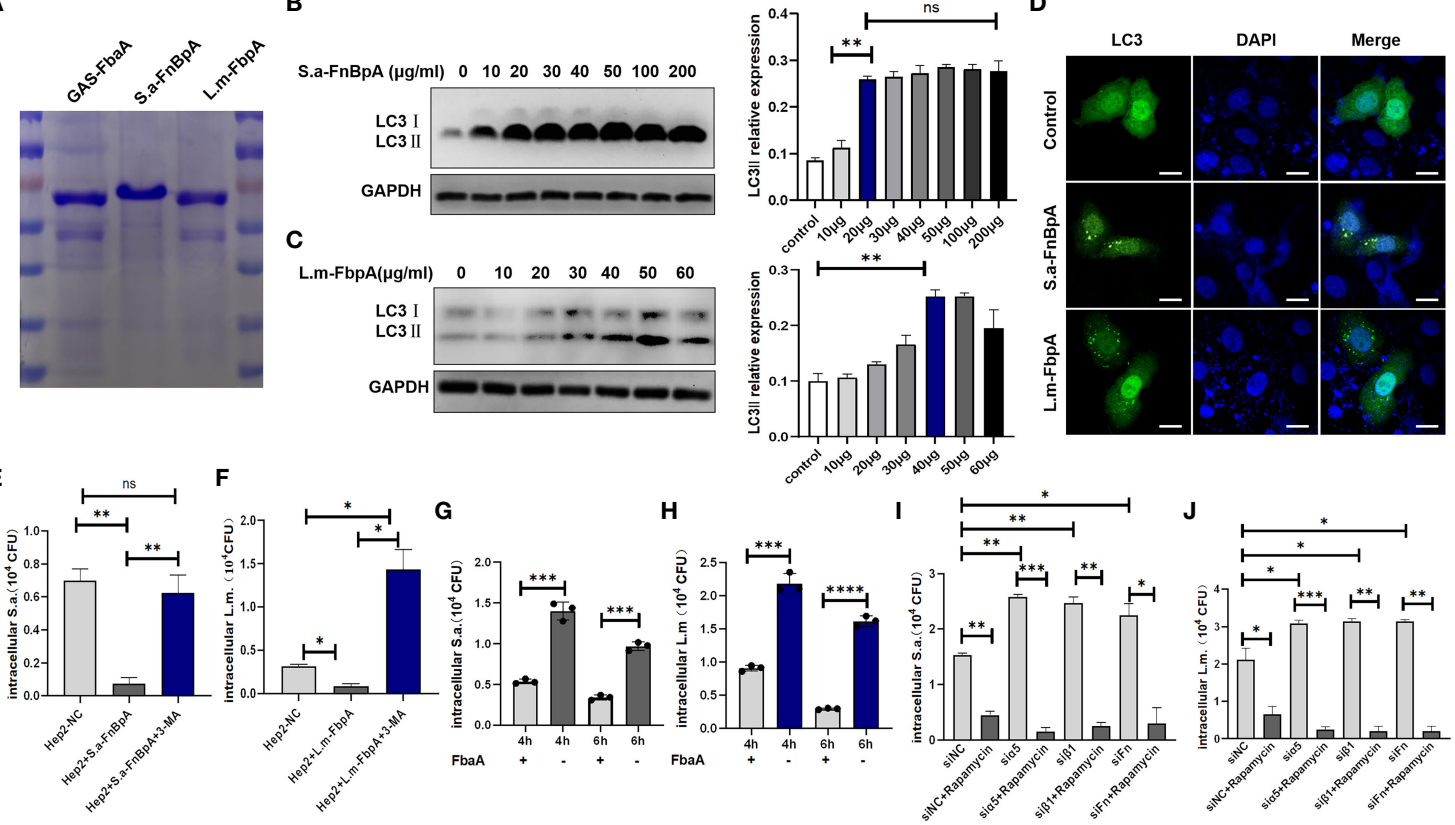
FnBp protein of common pathogens may trigger autophagy. **(A)** SDS-PAGE analysis of the expression and purification of FnBps in BL21 *E*. *coli* transfected with recombinant plasmids pET28a (+)/GAS-FbaA, pET28a (+)/S.a-FnBpA or pET28a (+)/L.m-FbpA. **(B, C)** Western blot analysis of LC3II protein level in cells stimulated with S.a-FnBpA and L.m-FbpA in the condition that FnBps have no toxic effect on cell growth. N = 3 repeats and the quantifications are means ± SD fold-change relative to control conditions for a given protein after normalization with GAPDH. **(D)** After EGFP-LC3- overexpressing cells were stimulated with S.a-FnBpA (20 μg/ml) or L.m-FbpA (40 μg/ml) for 6 h, confocal microscopy imaging detected that LC3 protein with green fluorescence showed obvious spot-like aggregation morphology in the cytoplasm of stimulated groups. Scale bar, 25 μm. **(E, F)** Hep2 cells were pretreated with purified FnBps or FnBp combined with 3-MA (10 mM) for 0.5 h and then infected with pathogens. Intracellular viable bacterial CFUs were detected in cells. **(G, H)** GAS-FbaA was added to the culture supernatant of *S. aureus* or *L. monocytogenes* infected Hep2 cells for 6h, respectively, and intracellular viable bacteria were counted after killing the extracellular bacteria. **(I, J)** After pretreatment with Rapamycin, WT Hep2 cells and Fn- or integrin α5β1-knockdown Hep2 cells were infected with *S. aureus* or *L. monocytogenes* for 6 h, and the number of viable bacteria in different groups was measured. Unpaired *t* test was used for data analysis. *P<0.05, **P<0.01, ***P<0.005, ****P<0.001.

Our previous study has demonstrated that the GAS surface protein FbaA, a FnBp, is the key protein for inducing autophagy. Similarly, FnBps are also presented on the surface of various pathogens such as *S. aureus* and *L. monocytogenes* (Osanai et al., 2013; Foster, 2016; Josse et al., 2017; Speziale and Pietrocola, 2020), namely S.a-FnBpA and L.m-FbpA, respectively. Then, we purified S.a-FnBpA and L.m-FbpA separately (**Figure 4A**) and at protein level we verified the ability of the two proteins in inducing autophagy after excluding their potential cytotoxicity on cell proliferation (**Supplementary Figure S1**). The results of Western blot showed that the protein level of LC3 II was significantly increased after cells were stimulated with the two proteins, respectively (**Figures 4B, C**). Furthermore, LC3 green fluorescence showed punctate aggregation following stimulation with FnBps in the overexpressing GFP-LC3 cells, indicating the occurrence of autophagy. (**Figure 4D**). Subsequently, cells were pretreated with purified S.a-FnBpA or L.m-FbpA protein, or FnBps plus 3-MA (PI3K inhibitor, autophagy inhibitor), or PBS as control for 30min, respectively, following infection by *S. aureus* or *L. monocytogenes*, and the results showed that FnBps stimulation resulted in a significant decrease in the number of viable bacteria. However, after adding 3-MA, the number of viable bacteria increased sharply owing to the inhibition of autophagy (**Figures 4E, F**), indicating that FnBp-induced autophagy was beneficial to eliminating the invading bacteria. Interestingly, after FbaA of GAS was applied to *S. aureus* or *L. monocytogenes*-infected epithelial cells, the number of intracellular invading bacteria decreased significantly, suggesting that the ability of FnBp to facilitate the elimination of intracellular invading bacteria by inducing autophagy may be universal (**Figures 4G, H**). Since integrin α5β1 acts as a receptor for the Fn-FnBp complex, knocking down Fn protein, integrin α5 chain or integrin β1 chain results in significantly increased number of intracellular viable bacteria, which was, however, decreased following the treatment of autophagy agonist Rapamycin (**Figures 4I, J**), suggesting that these three proteins are indispensable in the process of FnBp-mediated autophagy and that FnBps on the surface of the two bacteria can trigger the autophagy.

### Integrin α5β1 is a common receptor for Fn-FnBps complex in inducing autophagy

**Figure 5:**
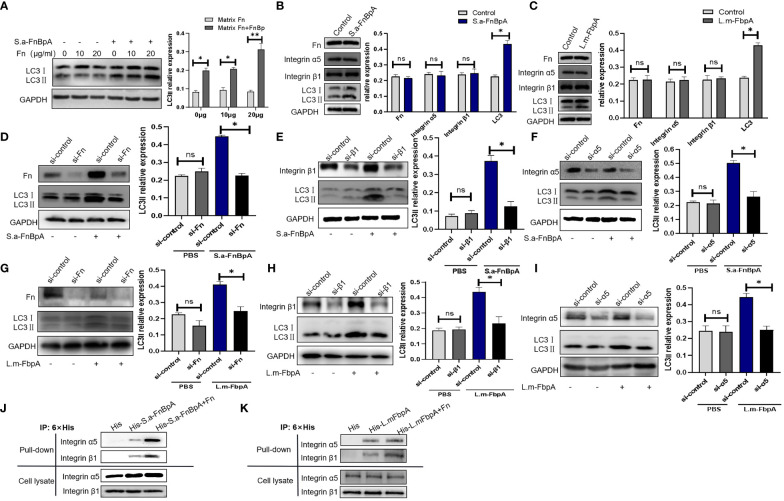
FnBps induce autophagy though the FnBp-Fn-integrin α5β1 axis. **(A)** Hep2 cells were stimulated by different concentrations of Fn protein with or without S.a-FnBpA protein. Thereafter, LC3II protein level was detected by Western blot. **(B, C)** Hep2 cells were stimulated by using S.a-FnBpA (20μg/ml) and L.m-FbpA (40μg/ml) for 6 h, and then the protein levels of Fn, integrin α5β1, or LC3II were detected by Western blot. **(D-I)** An siRNA was performed to knock down Fn protein, integrin α5 chain or β1 chain of Hep2 cells, respectively. After conditioning treatment, S.a-FnBpA or L.m-FbpA protein was added into cell culture supernatant of different experimental groups. The knockdown effect of Fn protein and integrin α5β1 was determined by Western Blot; the protein level of intracellular LC3II was detected as well. For the above WB experiments, n = 3 repeats and the quantifications are means ± SD fold-change relative to control conditions for a given protein after normalization with GAPDH. **(J, K)** Pull-down assays were performed to detect the binding ability of FnBps to integrin α5β1 with/without Fn protein. Unpaired *t* test was used for data analysis. *P<0.05, **P<0.01.

In order to exclude the possibility that Fn protein alone affects autophagy level, Hep2 cells were treated with Fn alone (0µg/ml, 10µg/ml, 20µg/ml) or Fn and S.a-FnBpA. The results showed that different doses of Fn protein could not induce changes in the protein level of LC3II, which was increased only when S. a-FnBpA and Fn protein were introduced for co-stimulation in a dose-dependent way (**Figure 5A**). Meanwhile, S.a-FnBpA or L.m-FbpA did not induce changes in the protein levels of Fn or integrin α5β1 when they triggered autophagy (**Figures 5B, C**). Subsequently, in order to verify whether FnBp induced autophagy is achieved through the FnBp-Fn-α5β1 axis, Fn protein, integrin α5 subunit or β1 subunit was knocked down by using siRNA in Hep2 cells, which were then stimulated by S.a-FnBpA or L.m-FbpA. After being treated by FnBp, results showed that the protein level of LC3II in knockdown Fn or integrin α5 or β1 groups was significantly lower than that in the control group (**Figures 5D-I**), suggesting that integrin α5 chain, β1 chain and Fn protein are indispensable links in the autophagy initiated by FnBps. In order to further clarify the combined effects of these three factors in inducing autophagy, pull-down experiments were performed. Results showed that FnBps were more capable of binding to integrin α5β1 than the control group when Fn protein was present (**Figures 5J, K**). Taken together, FnBps could induce autophagy through the FnBp-Fn-integrin α5β1 axis, while matrix protein Fn could enhance the binding ability between FnBps and integrin α5β1.

### S100A8 is a key downstream molecule involved in autophagy via FnBp-Fn-integrin α5β1 axis

**Figure 6:**
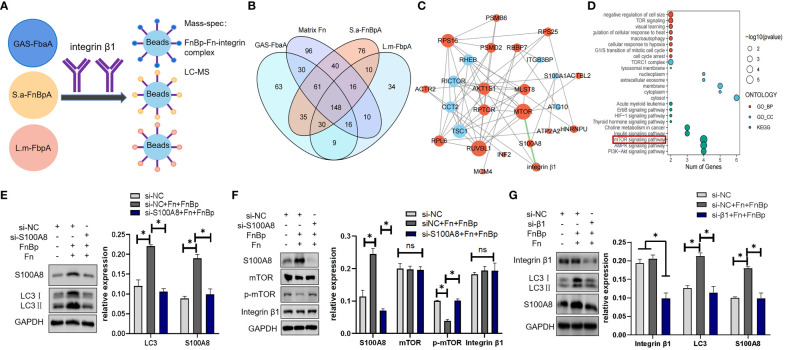
S100A8 is a key downstream protein in regulating autophagy initiated by the receptor integrin α5β1. **(A)** In the presence of Fn protein, Hep2 cells were stimulated with GAS-FbaA, S.a-FnBpA, and L.m-FbpA, respectively, and Co-IP was performed with anti**-**integrin β1 mAb. Bound proteins of each group were collected for mass spectrometry analysis. **(B)** Based on protein mass spectrometry data analysis, we took the intersection of three FnBps groups and removed the data of individual Fn protein groups at this intersection, ultimately obtaining 30 target proteins. **(C, D)** Based on bioinformatics analysis, S100A8 and PSMD2 were present in these 30 potential proteins and enriched in the mTOR pathway. **(E, F)** Next, siRNA was used to knock down S100A8, and the protein levels of LC3, integrin β1, mTOR and p-mTOR were measured by Western blot under the stimulation of different groups. **(G)** After knocking down integrin β1 by siRNA, the expression of S100A8 and LC3 was detected by Western blot. For the above WB experiments, n = 3 repeats and the quantifications are means ± SD fold-change relative to control conditions for a given protein after normalization with GAPDH. Unpaired *t* test was used for data analysis. *P<0.05.

The above experiments showed that FnBps could trigger autophagy through the FnBp-Fn-integrin α5β1 axis. The FbaA protein, a FnBp of GAS, initiates autophagy through the mTOR signaling pathway (Wang et al., 2020). Since S.a-FnBpA of *S. aureus* and L.m-FbpA of *L. monocytogenes* both belong to the group of Fn-binding proteins, we asked if they initiate autophagy through the same signaling pathway and if other molecules are involved between integrin α5β1 and mTOR. Several research groups have identified the β1 subunit as the key signal transduction molecule in integrin α5β1 (Gingras and Ginsberg, 2020; Kadry and Calderwood, 2020; Torres-Gomez et al., 2020). Based on this, IP and mass spectrometry (MS) were performed to explore the potential downstream molecules of integrin β1 chain, and 30 proteins with high expression were screened out (**Figures 6A, B**). On the basis of bioinformatics analysis, S100A8 and PSMD2 became the foci of our attention (**Figure 6C**), and KEGG enrichment analysis showed that these proteins played a vital role in the mTOR pathway (**Figure 6D**). However, knockdown of PSMD2 had no effect on the expression of autophagy marker proteins in the cells. Only knocking down S100A8 could reduce LC3II protein level and increase the level p62 protein (**Supplementary Figure S2**), suggesting that FnBp-stimulated autophagy could be regulated by S100A8, which belongs to the S100 calcium-binding protein family and can be expressed in epithelial cells, neutrophils and monocytes. It also has certain antibacterial and anti-infective activities (Li et al., 2021b). Besides detecting the level of LC3II protein in S100A8-knockdown cells stimulated with FnBps and Fn (**Figure 6E**), we also detected the level of integrin β1 and mTOR, and compared the levels in the three groups. The results showed that there was no significant difference between integrin β1 protein level and mTOR level after S100A8 was knocked down, while phosphorylation level of mTOR was significantly increased, indicating that S100A8 may be located between integrin β1 and mTOR and could down-regulate the phosphorylation level of mTOR (**Figure 6F**). Further, when we knocked down integrin β1 under the same conditions as those noted above, results confirmed that the expression levels of S100A8 and LC3II proteins were remarkably decreased compared to the non-knockdown group, further supporting S100A8 as a key regulatory downstream molecule of integrin β1 in inducing autophagy by downregulating mTOR phosphorylation (**Figure 6G**).

## Discussion

In the last few decades, different research groups have found that multiple bacteria, including traditional intracellular and extracellular bacteria, such as GAS, *S. aureus*, *L. monocytogenes*, TB, BCG, NME and Ype, can enter epithelial or endothelial cells to escape phagocytosis by immune cells and/or antibiotic attack, causing chronic infections and constant threat to human health (Vázquez-Boland et al., 2001; Giese et al., 2011; Lu et al., 2017). Traditional antibiotic therapy could not meet the needs of clinical treatment, making host-directed therapies (HDTs) much more crucial (Kaufmann et al., 2018; Segala et al., 2021).

Increasingly, evidence has shown that autophagy plays an essential role in both innate and adaptive immunity (Deretic et al., 2013; Deretic and Levine, 2018). Some invasive bacteria, such as TB*, Salmonella enteritidis* (S.E)*, Salmonella typhimurium* (Sty) and *Pseudomonas aeruginosa* (Pae), can be further eliminated by autophagy (Birmingham and Brumell, 2006; Jiao et al., 2020; Siregar et al., 2022; Ji et al., 2023). However, some pathogens have evolved survival strategies in their favor, using autophagy for intracellular reproduction. This process has been achieved through LAP (Prajsnar et al., 2021). Therefore, we explored the exact mechanism of mutual recognition and induction of autophagy between pathogen and host cells, in which host cells can survive but bacteria cannot.

In our previous study, we found M1 GAS, one of the most common extracellular pathogens that invades epithelial cells through its surface protein FbaA, can initiate epithelial autophagy during this process. Subsequently, we verified that integrin α5β1 on epithelial cells was also the receptor for FbaA of M1 GAS in inducing autophagy (Wang et al., 2020). Li et al. found that integrin β1 is a novel regulator of autophagy and apoptosis in *Helicobacter pylori* (HP) infection (Li et al., 2021a). Hynes et al. discovered the integrin family in 1987, which has become one of the most studied cell adhesion receptors (Hynes, 2002). The integrin family is not only an effective therapeutic target against blood clots and inflammation, but also a receptor for many viruses and bacteria (Nolte et al., 2021; Schumacher et al., 2021; Pang et al., 2023). Especially, as noted above, integrin α5β1 is a receptor for various FnBp^+^ bacteria, such as TB, BCG, NME, NGO, Bbu, Ype, Llactis, GAS, *S. aureus* and *L. monocytogenes.*

**Figure 7:**
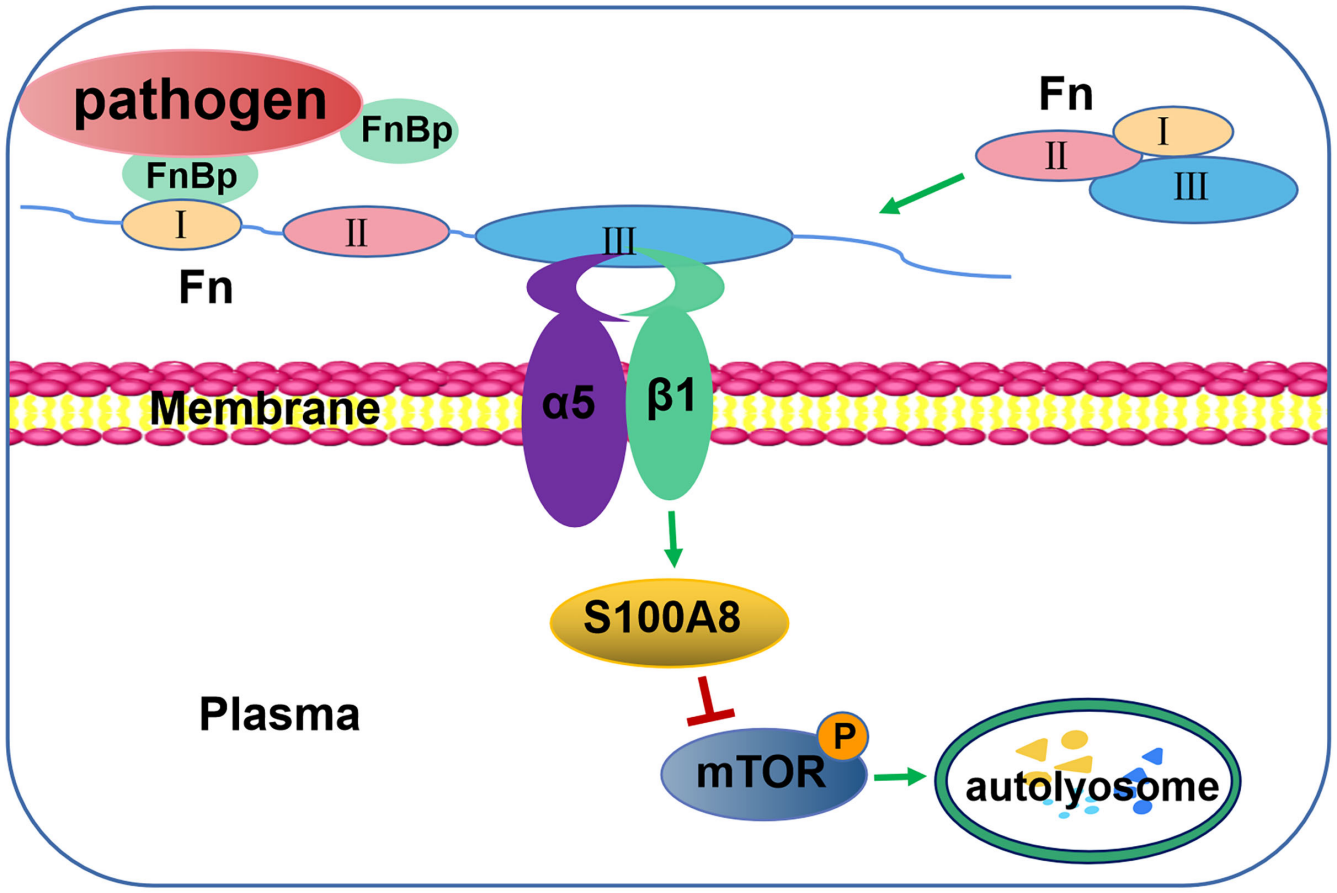
Schematic illustration of autophagy induction by FnBp through interaction between Fn and integrin α5β1 via the mTOR pathway. The binding of FnBp, Fn, and integrin α5β1 leads to the expression and activation of S100A8, thereby downregulating mTOR and, in turn, upregulating autophagy.

FbaA expressed on the surface of GAS belongs to the family of Fn-binding proteins. Fn, a large molecular glycoprotein with a molecular weight of 230kDa, is an indispensable part of integrin-induced autophagy. Fn, produce by a variety of cells, is widely distributed in human plasma. As shown in **Supplementary Figure S3**, Fn has a modular structure composed of types I, II and III (FNI, FNII and FNIII), which form different functional domains (Niemann et al., 2021). The N-terminal domain (NTD) consisting of five FNI-type modules is adjacent to the gelatin-binding domain (GBD) (Erat et al., 2010). Some pathogens can also bind the NTD or GBD of Fn through their fibronectin-binding protein (FnBp) (**Supplementary Figure S3**) (Talay et al., 2000; Schwarz-Linek et al., 2003; Singh et al., 2010). Normally, Fn is a monomer in a folded state in plasma. But, when FnBp binds to the FNI region of Fn, it will induce structural changes of Fn and expose the FNIII region for strong integrin binding (**Figure 7**) (Singh et al., 2010). In addition to M1 GAS, many other clinically common bacteria also express FnBps, such as TB, *S. aureus* and *L. monocytogenes* (Speziale et al., 2019).

Here, we selected *S. aureus* and *L. monocytogenes*, common infection-inducing pathogens in clinical treatment, as conventional extracellular and intracellular FnBp^+^ bacteria representatives, respectively. Some studies have reported on the relationship between the two and autophagy. Tomasz K. et al. found that autophagy can provide an intracellular replication niche for *S. aureus* within neutrophils (Prajsnar et al., 2021). Meanwhile, Josie F. et al., in the same lab, found that the selective autophagy receptor Sqstm1/p62 in neutrophils could target and degrade *S. aureus* intracellularly (Gibson et al., 2021). As for the relationship between *L. monocytogenes* and autophagy, Yuan et al. pointed out that *L. monocytogenes* is the target of autophagy degradation in mouse embryonic fibroblasts (MEF) during primary infection (Py et al., 2007), while they also found that *L. monocytogenes* can evade autophagy by expressing bacterial phospholipase. In our preliminary experiment, we found that if the amount of invading bacteria is overloaded (MOI≥100), the number of bacteria (either *S. aureus* or *L. monocytogenes*) within the cells didn’t decrease with time passing (data not shown). Bacteria may rely on their own toxins to escape from autophagosome and damage host cells (Watkins and Unnikrishnan, 2020). However, when cells were treated with fewer amount of bacteria (MOI ≤ 10), the invading bacteria would be removed easily by autophagy as the infection time prolongs and *L. monocytogenes*, a traditional intracellular pathogen, is no exception (**Supplementary Figure S4**), which may be the main mechanism for non-immune cells to remove intracellular bacteria. Here, in addition to confirming the ability of *S.aureus*, *L. monocytogenes* and BCG (**Supplementary Figure S5**) to induce autophagy in epithelial cells, we purified the major FnBp proteins of *S. aureus* and *L. monocytogenes*, S.a-FnBpA and L.m-FbpA, respectively (Osanai et al., 2013; Speziale and Pietrocola, 2020). Interestingly, after excluding the underlying effect of these proteins on cell proliferation, we found that S.a-FnBpA and L.m-FbpA could elicit autophagy in the presence of Fn. Furthermore, after *S. aureus* and *L. monocytogenes* infected epithelial Hep2 cells at low MOI, the number of invading bacteria were eliminated significantly in Hep2 cells stimulated with S.a-FnBpA, L.m-FbpA or GAS-FbaA, suggesting the universal function of FnBp in promoting removal of invading pathogens from cells by inducing autophagy (**Figures 4E-H**). Accordingly, this effect of inducing autophagy and eliminating invading pathogens could be counteracted by autophagy inhibitor 3-MA (PI3K inhibitor) (**Figures 4E, F**). Since autophagy was significantly attenuated after Atg5 knockdown, Atg5-knockdown Hep2 cells were used as an autophagy-deficient cell model. When infected with same pathogens, the level of autophagy was weakened in Atg5-knockdown cells. Meanwhile, *S. aureus* and *L. monocytogenes* were also removed from the lung in WT C57BL/6N mice, but not in Atg5 (KO) mice. These data revealed that autophagy mediated by FnBp is the mechanism of eliminating partial common clinical FnBp^+^ pathogens from host.

Further, siRNA was performed to knock down the protein level of Fn, α5 subunit or β1 subunit, respectively, in Hep2 cells, which then were treated with FnBp. The results showed that the protein level of LC3II protein in the siRNA interference group was significantly lower than that in the untreated group. Subsequently, pull-down assay showed that the protein level of integrin α5 or β1 chain bound by FnBp in the presence of Fn protein was significantly higher than that of FnBp alone. These results showed that FnBps can induce autophagy and play their role through the FnBp-Fn-integrin α5β1 axis. By MS and bioinformatics analysis, S100A8 and PSMD2 were identified as potential regulatory downstream molecules of integrin α5β1, both targeting mTOR phosphorylation, a well-known negative regulator of autophagy. However, knockdown of PSMD2 did not affect autophagy mediated by FnBp, while knockdown of S100A8 did, suggesting that S100A8 may be the key molecule in regulating the interaction between integrin α5β1 and mTOR. S100A8 belongs to the S100 protein family, which is associated with cell growth, differentiation and replication (Mondet et al., 2021). S100A8 is a low molecular weight protein, about 12kDa, composed of 93 amino acids. It is also known as myeloid associated protein-8 (MRP-8) and calgranin A (Basso et al., 2014), expressing on the surface of monocytes, macrophages and non-professional phagocytes, such as epithelial cells, but it is absent in lymphocytes (Bartoloni et al., 2019; Zhong et al., 2020; Mondet et al., 2021). After S100A8 was knocked down, we found that the phosphorylation level of mTOR was significantly increased, while the level of autophagy marker protein LC3 was significantly reduced. Meanwhile, the protein level of integrin β1 remained basically stable. Interestingly, when integrin β1 is knocked down, S100A8 protein levels are also reduced, suggesting that S100A8, as a key downstream molecule of integrin β1, plays a crucial role in inducing autophagy by downregulating mTOR phosphorylation, thus upregulating autophagy.

In summary, this study reveals a novel model, FnBp-induced autophagy, that facilitates the elimination of invading pathogens from the host, even though FnBp^+^ bacteria can invade cells by FnBps to evade immune attack from immune cells. We confirmed that integrin α5β1-initiated autophagy is a common event in epithelial cells following interaction with Fn and FnBp for host defense against FnBp^+^ pathogen infection, and we are the first to reveal the key molecule of the integrin β1 chain, S100A8, which is highly expressed following activation of integrin α5β1. S100A8 also downregulates mTOR phosphorylation, which, in turn, regulates autophagy so as to promote the classical autophagy pathway (**Figure 7**). This study provides new insights into the interaction pattern between pathogens and hosts and identifies the target protein responsible for regulating autophagy. We hope that the future studies will produce new strategies for developing treatment for drug-resistant bacteria infection.

## Data availability statement

The original contributions presented in the study are included in the article/**Supplementary Material**. Further inquiries can be directed to the corresponding authors.

## Ethics statement

Ethical approval was not required for the studies on humans in accordance with the local legislation and institutional requirements because only commercially available established cell lines were used. The animal study was approved by HeBei Medical University Laboratory Animal Ethical and Welfare Committee. The study was conducted in accordance with the local legislation and institutional requirements.

## Author contributions

MM: Writing – original draft. JiacW: Writing – original draft. HL: Writing – original draft. JiaoW: Writing – original draft. XW: Writing – original draft. ML: Writing – original draft. XG: Writing – original draft. WL: Writing – original draft. CM: Writing – review & editing. LW: Writing – review & editing.

## References

[B1] Bartoloni E. Alunno A. Cafaro G. Valentini V. Bistoni O. Bonifacio A.. (2019). Subclinical atherosclerosis in primary sjögren's syndrome: does inflammation matter? Front. In Immunol. 10, 817. doi: 10.3389/fimmu.2019.00817 31110500 PMC6499202

[B2] Basso D. Bozzato D. Padoan A. Moz S. Zambon C. Fogar P.. (2014). Inflammation and pancreatic cancer: molecular and functional interactions between S100a8, S100a9, nt-S100a8 and tgfβ1. Cell Communication And Signaling Ccs 12, 20. doi: 10.1186/1478-811X-12-20 24670043 PMC4108065

[B3] Birmingham C. Brumell J. (2006). Autophagy recognizes intracellular salmonella enterica serovar typhimurium in damaged vacuoles. Autophagy 2, 156–158. doi: 10.4161/auto.2825 16874057

[B4] Caire R. Audoux E. Thomas M. Dalix E. Peyron A. Rodriguez K.. (2022). Yap promotes cell-autonomous immune responses to tackle intracellular staphylococcus aureus *in vitro* . Nat. Commun. 13 (1), 6995. doi: 10.1038/s41467-022-34432-0 36384856 PMC9669043

[B5] Deretic V. Levine B. (2009). Autophagy, immunity, and microbial adaptations. Cell Host Microbe 5, 527–549. doi: 10.1016/j.chom.2009.05.016 19527881 PMC2720763

[B6] Deretic V. Levine B. (2018). Autophagy balances inflammation in innate immunity. Autophagy 14, 243–251. doi: 10.1080/15548627.2017.1402992 29165043 PMC5902214

[B7] Deretic V. Saitoh T. Akira S. (2013). Autophagy in infection, inflammation and immunity. Nat. Rev. Immunol. 13, 722–737. doi: 10.1038/nri3532 24064518 PMC5340150

[B8] Erat M. Schwarz-Linek U. Pickford A. Farndale R. Campbell I. Vakonakis I. (2010). Implications for collagen binding from the crystallographic structure of fibronectin 6fni1-2fnii7fni. J. Of Biol. Chem. 285, 33764–33770. doi: 10.1074/jbc.M110.139394 20739283 PMC2962475

[B9] Foster T. (2016). The remarkably multifunctional fibronectin binding proteins of staphylococcus aureus. Eur. J. Of Clin. Microbiol. Infect. Dis. Off. Publ. Of Eur. Soc. Of Clin. Microbiol. 35, 1923–1931. doi: 10.1007/s10096-016-2763-0 27604831

[B10] Gibson J. Prajsnar T. Hill C. Tooke A. Serba J. Tonge R.. (2021). Staphylococcus aureusneutrophils use selective autophagy receptor sqstm1/P62 to target for degradation in zebrafish. Autophagy 17, 1448–1457. doi: 10.1080/15548627.2020.1765521 32559122 PMC8204994

[B11] Giese B. Glowinski F. Paprotka K. Dittmann S. Steiner T. Sinha B.. (2011). Expression of Δ-toxin by staphylococcus aureus mediates escape from phago-endosomes of human epithelial and endothelial cells in the presence of Β-toxin. Cell. Microbiol. 13, 316–329. doi: 10.1111/j.1462-5822.2010.01538.x 20946243

[B12] Gingras A. Ginsberg M. (2020). Signal transduction: physical deformation of the membrane activates integrins. Curr. Biol. Cb 30, R397–R400. doi: 10.1016/j.cub.2020.02.068 32369751

[B13] Hynes R. (2002). Integrins: bidirectional, allosteric signaling machines. Cell 110, 673–687. doi: 10.1016/S0092-8674(02)00971-6 12297042

[B14] Ji X. Jin P. Yu P. Wang P. (2023). Autophagy ameliorates pseudomonas aeruginosa-infected diabetic wounds by regulating the toll-like receptor 4/myeloid differentiation factor 88 pathway. Wound Repair And Regeneration Off. Publ. Of Wound Healing Soc. [And] Eur. Tissue Repair Soc. 31, 305–320. doi: 10.1111/wrr.13074 36879445

[B15] Jiao Y. Zhang Y. Lin Z. Lu R. Xia Y. Meng C.. (2020). Salmonella enteritidis effector avra suppresses autophagy by reducing beclin-1 protein. Front. Immunol. 11, 686. doi: 10.3389/fimmu.2020.00686 32362899 PMC7181453

[B16] Josse J. Laurent F. Diot A. (2017). Staphylococcal adhesion and host cell invasion: fibronectin-binding and other mechanisms. Front. In Microbiol. 8, 2433. doi: 10.3389/fmicb.2017.02433 PMC5723312 29259603

[B17] Kadry Y. Calderwood D. (2020). Chapter 22: structural and signaling functions of integrins. Biochim. Et Biophys. Acta Biomembranes 1862, 183206. doi: 10.1016/j.bbamem.2020.183206 PMC7063833 31991120

[B18] Kaufmann S. Dorhoi A. Hotchkiss R. Bartenschlager R. (2018). Host-directed therapies for bacterial and viral infections. Nat. Rev. Drug Discovery 17, 35–56. doi: 10.1038/nrd.2017.162 28935918 PMC7097079

[B19] Kemper L. Hensel A. (2023). Campylobacter jejuni: targeting host cells, adhesion, invasion, and survival. Appl. Microbiol. And Biotechnol. 107, 2725–2754. doi: 10.1007/s00253-023-12456-w 36941439 PMC10027602

[B20] Krakauer T. (2019). Inflammasomes, autophagy, and cell death: the trinity of innate host defense against intracellular bacteria. Med. Inflamm. 2019, 2471215. doi: 10.1155/2019/2471215 PMC6341260 30728749

[B21] Kuo C. Hansen M. Troemel E. (2018). Autophagy and innate immunity: insights from invertebrate model organisms. Autophagy 14, 233–242. doi: 10.1080/15548627.2017.1389824 29130360 PMC5902216

[B22] Kwon D. Song H. (2018). A structural view of xenophagy, A battle between host and microbes. Molecules And Cells 41, 27–34. doi: 10.14348/molcells.2018.2274 29370690 PMC5792709

[B23] Li B. Rong Q. Du Y. Zhang R. Li J. Tong X.. (2021a). Regulation of Β1-integrin in autophagy and apoptosis of gastric epithelial cells infected with helicobacter pylori. World J. Of Microbiol. Biotechnol. 38, 12. doi: 10.1007/s11274-021-03199-9 34873651

[B24] Li X. Cao G. Yang H. Zhi D. Li L. Wang D.. (2021b). S100a8 expression in oviduct mucosal epithelial cells is regulated by estrogen and affects mucosal immune homeostasis. PloS One 16, E0260188. doi: 10.1371/journal.pone.0260188 34793556 PMC8601440

[B25] Lu S. Kawabata T. Cheng Y. Omori H. Hamasaki M. Kusaba T.. (2017). Endothelial cells are intrinsically defective in xenophagy of streptococcus pyogenes. PloS Pathog. 13, E1006444. doi: 10.1371/journal.ppat.1006444 28683091 PMC5500369

[B26] Ma C. Li C. Wang X. Zeng R. Yin X. Feng H.. (2009). Similar ability of fbaa with M protein to elicit protective immunity against group A streptococcus challenge in mice. Cell. Mol. Immunol. 6, 73–77. doi: 10.1038/cmi.2009.10 19254483 PMC4002543

[B27] Mitchell G. Cheng M. Chen C. Nguyen B. Whiteley A. Kianian S.. (2018). Listeria monocytogenes triggers noncanonical autophagy upon phagocytosis, but avoids subsequent growth-restricting xenophagy. Proc. Natl. Acad. Of Sci. U. S. A. 115, E210–E217. doi: 10.1073/pnas.1716055115 29279409 PMC5777066

[B28] Mondet J. Chevalier S. Mossuz P. (2021). Pathogenic roles of S100a8 and S100a9 proteins in acute myeloid and lymphoid leukemia: clinical and therapeutic impacts. Molecules (Basel Switzerland) 26 (5), 1323. doi: 10.3390/molecules26051323 33801279 PMC7958135

[B29] Nakatogawa H. (2020). Mechanisms governing autophagosome biogenesis. Nat. Rev. Mol. Cell Biol. 21, 439–458. doi: 10.1038/s41580-020-0241-0 32372019

[B30] Neumann Y. Bruns S. Rohde M. Prajsnar T. Foster S. Schmitz I. (2016). Intracellular staphylococcus aureus eludes selective autophagy by activating A host cell kinase. Autophagy 12, 2069–2084. doi: 10.1080/15548627.2016.1226732 27629870 PMC5103350

[B31] Niemann S. Nguyen M. Eble J. Chasan A. Mrakovcic M. Böttcher R.. (2021). More is not always better-the double-headed role of fibronectin in staphylococcus aureus host cell invasion. Mbio 12, E0106221. doi: 10.1128/mBio.01062-21 34663090 PMC8524341

[B32] Nolte M. Nolte-'T Hoen E. Margadant C. (2021). Integrins control vesicular trafficking; new tricks for old dogs. Trends In Biochem. Sci. 46, 124–137. doi: 10.1016/j.tibs.2020.09.001 33020011 PMC7531435

[B33] Osanai A. Li S. Asano K. Sashinami H. Hu D. Nakane A. (2013). Fibronectin-binding protein, fbpa, is the adhesin responsible for pathogenesis of listeria monocytogenes infection. Microbiol. Immunol. 57, 253–262. doi: 10.1111/1348-0421.12030 23586629

[B34] Pang X. He X. Qiu Z. Zhang H. Xie R. Liu Z.. (2023). Targeting integrin pathways: mechanisms and advances in therapy. Signal Transduction Targeted Ther. 8, 1. doi: 10.1038/s41392-022-01259-6 PMC9805914 36588107

[B35] Patterson L. Byerly C. Mcbride J. (2021). Anaplasmataceae: dichotomous autophagic interplay for infection. Front. Immunol. 12, 642771. doi: 10.3389/fimmu.2021.642771 33912170 PMC8075259

[B36] Prajsnar T. Serba J. Dekker B. Gibson J. Masud S. Fleming A.. (2021). Staphylococcus aureusthe autophagic response to provides an intracellular niche in neutrophils. Autophagy 17, 888–902. doi: 10.1080/15548627.2020.1739443 32174246 PMC8078660

[B37] Py B. Lipinski M. Yuan J. (2007). Autophagy limits listeria monocytogenes intracellular growth in the early phase of primary infection. Autophagy 3, 117–125. doi: 10.4161/auto.3618 17204850

[B38] Rodrigues Lopes I. Alcantara L. Silva R. Josse J. Vega E. Cabrerizo A.. (2022). Microscopy-based phenotypic profiling of infection by staphylococcus aureus clinical isolates reveals intracellular lifestyle as A prevalent feature. Nat. Commun. 13, 7174. doi: 10.1038/s41467-022-34790-9 36418309 PMC9684519

[B39] Schille S. Crauwels P. Bohn R. Bagola K. Walther P. Van Zandbergen G. (2018). Lc3-associated phagocytosis in microbial pathogenesis. Int. J. Of Med. Microbiol. Ijmm 308, 228–236. doi: 10.1016/j.ijmm.2017.10.014 29169848

[B40] Schumacher S. Dedden D. Nunez R. Matoba K. Takagi J. Biertümpfel C.. (2021). Structural insights into integrin Αβ Opening by fibronectin ligand. Sci. Adv. 7 (19), eabe9716. doi: 10.1126/sciadv.abe9716 33962943 PMC8104898

[B41] Schwarz-Linek U. Werner J. Pickford A. Gurusiddappa S. Kim J. Pilka E.. (2003). Pathogenic bacteria attach to human fibronectin through A tandem beta-zipper. Nature 423, 177–181. doi: 10.1038/nature01589 12736686

[B42] Segala F. Bavaro D. Di Gennaro F. Salvati F. Marotta C. Saracino A.. (2021). Impact of sars-cov-2 epidemic on antimicrobial resistance: A literature review. Viruses 13 (11), 2110. doi: 10.3390/v13112110 34834917 PMC8624326

[B43] Shahnazari S. Brumell J. (2011). Mechanisms and consequences of bacterial targeting by the autophagy pathway. Curr. Opin. Microbiol. 14, 68–75. doi: 10.1016/j.mib.2010.11.001 21112809

[B44] Shibutani S. Yoshimori T. (2014). Autophagosome formation in response to intracellular bacterial invasion. Cell. Microbiol. 16, 1619–1626. doi: 10.1111/cmi.12357 25180443

[B45] Singh P. Carraher C. Schwarzbauer J. (2010). Assembly of fibronectin extracellular matrix. Annu. Rev. Cell Dev. Biol. 26, 397–419. doi: 10.1146/annurev-cellbio-100109-104020 20690820 PMC3628685

[B46] Siregar T. Prombutara P. Kanjanasirirat P. Kunkaew N. Tubsuwan A. Boonmee A.. (2022). The autophagy-resistant mycobacterium tuberculosis beijing strain upregulates katg to evade starvation-induced autophagic restriction. Pathog. Dis. 80 (1), ftac004. doi: 10.1093/femspd/ftac004 35038342 PMC8829027

[B47] Sorbara M. Foerster E. Tsalikis J. Abdel-Nour M. Mangiapane J. Sirluck-Schroeder I.. (2018). Complement C3 drives autophagy-dependent restriction of cyto-invasive bacteria. Cell Host Microbe 23, 644–652.E5. doi: 10.1016/j.chom.2018.04.008 29746835

[B48] Speziale P. Arciola C. Pietrocola G. (2019). Fibronectin and its role in human infective diseases. Cells 8 (12), 1516. doi: 10.3390/cells8121516 31779172 PMC6952806

[B49] Speziale P. Pietrocola G. (2020). Staphylococcus aureusthe multivalent role of fibronectin-binding proteins A and B (Fnbpa and fnbpb) of in host infections. Front. In Microbiol. 11, 2054. doi: 10.3389/fmicb.2020.02054 PMC7480013 32983039

[B50] Talay S. Zock A. Rohde M. Molinari G. Oggioni M. Pozzi G.. (2000). Co-operative binding of human fibronectin to sfbl protein triggers streptococcal invasion into respiratory epithelial cells. Cell. Microbiol. 2, 521–535. doi: 10.1046/j.1462-5822.2000.00076.x 11207605

[B51] Tang D. Kang R. Coyne C. Zeh H. Lotze M. (2012). Pamps and damps: signal 0s that spur autophagy and immunity. Immunol. Rev. 249, 158–175. doi: 10.1111/j.1600-065X.2012.01146.x 22889221 PMC3662247

[B52] Torres-Gomez A. Cabañas C. Lafuente E. (2020). Phagocytic integrins: activation and signaling. Front. In Immunol. 11, 738. doi: 10.3389/fimmu.2020.00738 32425937 PMC7203660

[B53] Vázquez-Boland J. Kuhn M. Berche P. Chakraborty T. Domínguez-Bernal G. Goebel W.. (2001). Listeria pathogenesis and molecular virulence determinants. Clin. Microbiol. Rev. 14, 584–640. doi: 10.1128/CMR.14.3.584-640.2001 11432815 PMC88991

[B54] Wang M. Fan Z. Han H. (2021). Staphylococcus aureusautophagy in infection. Front. Cell. Infection Microbiol. 11, 750222. doi: 10.3389/fcimb.2021.750222 PMC8529010 34692566

[B55] Wang J. Meng M. Li M. Guan X. Liu J. Gao X.. (2020). Streptococcusintegrin Α5β1, as A receptor of fibronectin, binds the fbaa protein of group A to initiate autophagy during infection. Mbio 11 (3), e00771–20. doi: 10.1128/mBio.00771-20 32518187 PMC7371361

[B56] Watkins K. Unnikrishnan M. (2020). Evasion of host defenses by intracellular staphylococcus aureus. Adv. Appl. Microbiol. 112, 105–141. doi: 10.1016/bs.aambs.2020.05.001 32762866

[B57] Xie X. Yang C. Duan C. Chen H. Zeng T. Huang S.. (2020). Advanced glycation end products reduce macrophage-mediated killing of staphylococcus aureus by arl8 upregulation and inhibition of autolysosome formation. Eur. J. Immunol. 50, 1174–1186. doi: 10.1002/eji.201948477 32250445

[B58] Zang H. Qian S. Li J. Zhou Y. Zhu Q. Cui L.. (2020). The effect of selenium on the autophagy of macrophage infected by staphylococcus aureus. Int. Immunopharmacol. 83, 106406. doi: 10.1016/j.intimp.2020.106406 32193097

[B59] Zhong X. Xie F. Chen L. Liu Z. Wang Q. (2020). S100a8 and S100a9 promote endothelial cell activation through the rage−Mediated mammalian target of rapamycin complex 2 pathway. Mol. Med. Rep. 22, 5293–5303. doi: 10.3892/mmr.2020.11595 33174028 PMC7646991

