# Supplementary material for: Eliminating the invading extracellular and intracellular FnBp+ bacteria from respiratory epithelial cells by autophagy mediated through FnBp-Fn-Integrin α5β1 axis
Source: Front Cell Infect Microbiol. 2024 Jan 9;13:1324727. doi: 10.3389/fcimb.2023.1324727 (PMC10803403; doi:10.3389/fcimb.2023.1324727)
Supplement: Supplementary file 1 [file DataSheet_1.docx]

Supplementary Material

##
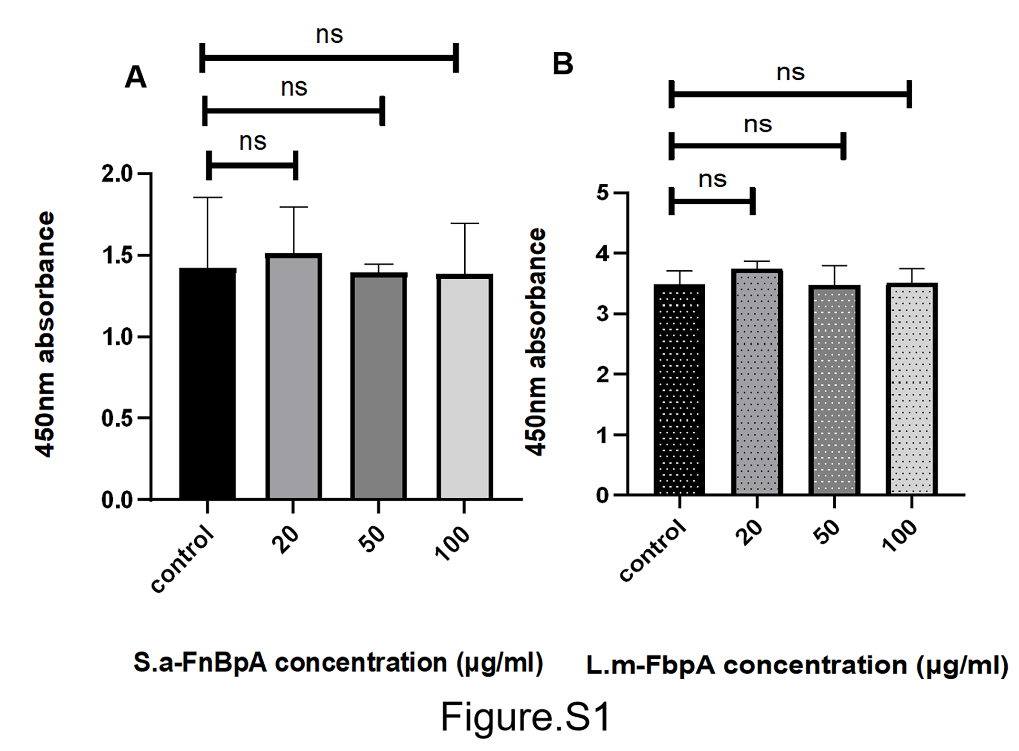
Supplementary Figures

**Supplementary Figure 1.** **FnBps have no potential cytotoxicity.**

(A-B) Hep2 epithelial cells were stimulated with S.a-FnBpA or L.m-FbpA protein in gradient concentrations. CCK8 assay was performed to determine possible cytotoxicity, while FnBps had no toxic effect on cells at various stimulation concentrations.


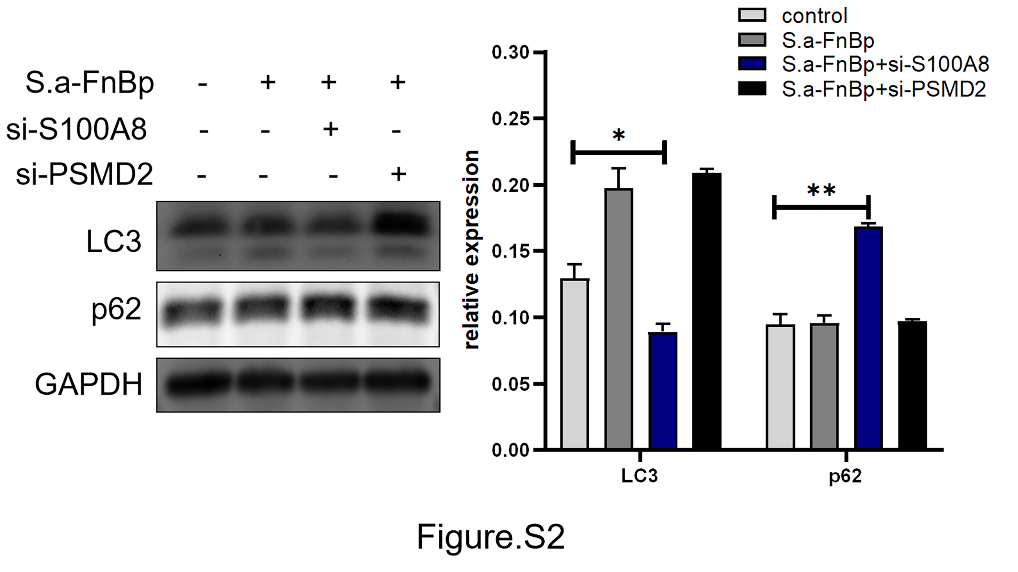


**Supplementary Figure 2.** **Knockdown of PSMD2 did not affect FnBp-induced autophagy.**

The siRNA was used to knock down PMSD2 and S100A8 in Hep2 epithelial cells, respectively. After pretreatment, FnBp was added to the cell culture supernatant of different experimental groups, and then the total cell protein of each group was collected for Western blot.


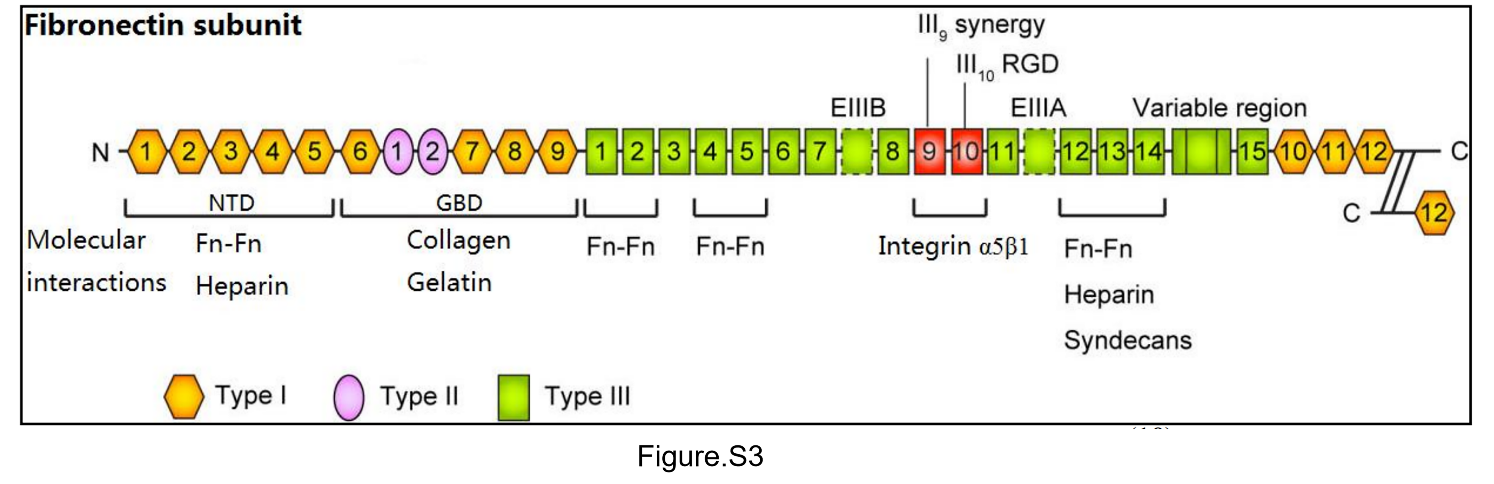


**Supplementary Figure 3.** **Diagram of the structure and functional domains of Fn protein^[1]^**.


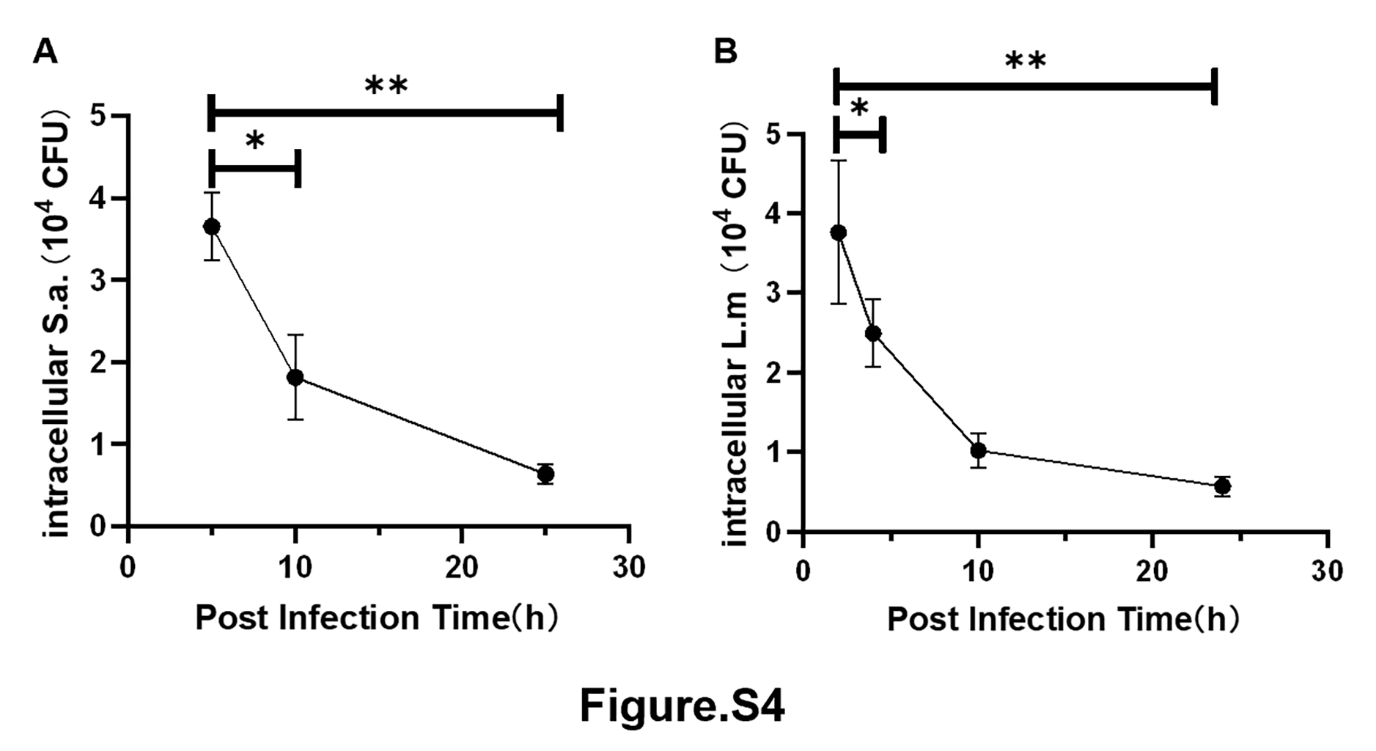


**Supplementary Figure 4. Amount of intracellular viable bacteria decreased with time gradient in condition of less MOI infection in respiratory epithelial cells.**

(A-B) Under the condition of MOI=10, *S. aureus* or *L. monocytogenes* were performed to infect the respiratory epithelial cell Hep2. Infected cells were collected at different time points, intracellular viable bacteria were counted after killing the extracellular bacteria.


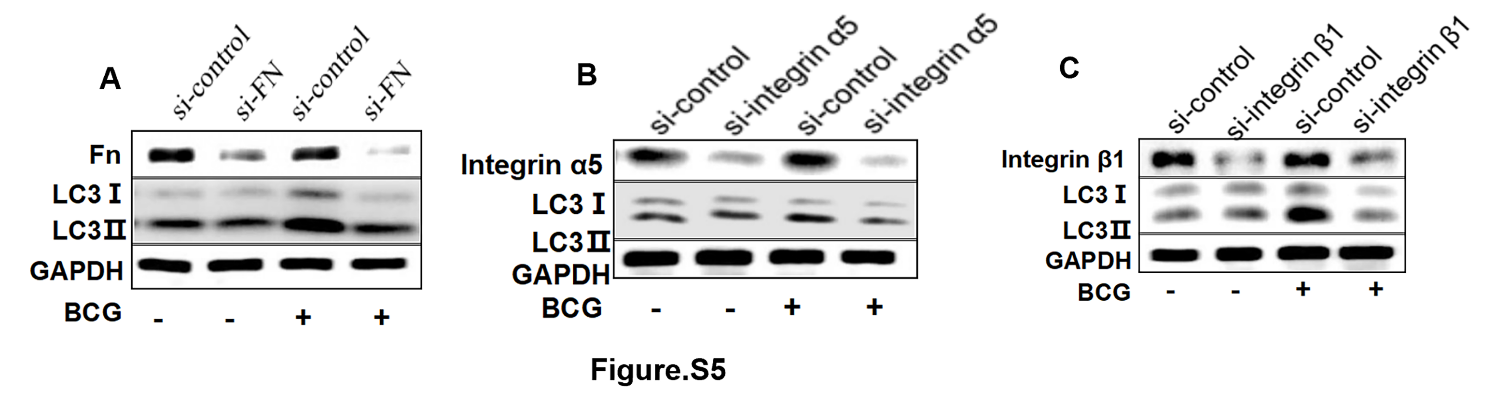
**Supplementary Figure 5.** **BCG, another representative of intracellular FnBp^+^ bacteria, induce autophagy through binding Fn and integrin α5β1.**

(A-C) SiRNA was performed to knock down Fn protein, integrin α5 chain or β1 chain of Hep2 cells, respectively. After conditioning treatment, BCG was added into cell culture supernatant of different experimental groups. The knockdown effect of Fn protein and integrin α5β1 was determined by Western Blot; the protein level of intracellular LC3II was detected as well.

[1] Singh P, Carraher C, Schwarzbauer J. Assembly of fibronectin extracellular matrix[J]. Annual review of cell and developmental biology, 2010, 26:397-419.
